# Can Doll therapy preserve or promote attachment in people with cognitive, behavioral, and emotional problems? A pilot study in institutionalized patients with dementia

**DOI:** 10.3389/fpsyg.2014.00342

**Published:** 2014-04-21

**Authors:** Rita Pezzati, Valentina Molteni, Marco Bani, Carmen Settanta, Maria Grazia Di Maggio, Ivan Villa, Barbara Poletti, Rita B. Ardito

**Affiliations:** ^1^University of Applied Sciences and Arts of Southern SwitzerlandManno, Switzerland; ^2^Centro Terapia CognitivaComo, Italy; ^3^Istituti Riuniti Airoldi e Muzzi OnlusLecco, Italy; ^4^Center for Cognitive Science, Department of Psychology, University of TurinTurin, Italy; ^5^Department of Neurology and Laboratory of Neuroscience, Istituto di Ricovero e Cura a Carattere Scientifico Istituto Auxologico ItalianoMilan, Italy

**Keywords:** Alzheimer’s disease, attachment, behavioral problems, caregiving, dementia, Doll therapy, emotional problems, exploration

## Abstract

Doll therapy is a non-pharmacological intervention aimed at reducing behavioral and psychological disorders in institutionalized patients with dementia. This therapy as a care tool has been integrated into the context of long-term care institutions, in which the need to find solutions to cognitive, behavioral and emotional problems showed by people with dementia meets the primary objective of developing good care practices focusing on patients and their needs. In the present work we adopt the Bowlby’s theory of attachment to investigate the effectiveness of Doll therapy. The hypothesis that we here propose is that the emotional experience of the person with dementia during Doll therapy activates caregiving and exploration systems together with the attachment one. To test this hypothesis we compared institutionalized patients with dementia undergoing Doll therapy with a control group and assessed measures of the relational dimension with the environment, such as gaze direction, behaviors of exploration, and behaviors of caregiving. We used an experimental protocol consisting of 10 non-consecutive sessions structured with the goal of recreating a situation of (1) separation from a known figure and (2) interaction with the environment in order to partially recreate the prototypical phases of the “Strange situation.” All sessions were videotaped and analyzed through an observational grid. Results support the effectiveness of Doll therapy in promoting and maintaining the affective-relational dimension of attachment-caregiving and the attentive dimension of exploration in patients with advanced stage of dementia. Thus, our results suggest that the use of Doll therapy promotes clinically significant improvements in the ability to relate with the surrounding world. This may be important for managing and caring for patients with dementia in institutionalized context.

## INTRODUCTION

In nursing homes, the need to prevent and manage behavioral and psychological disorders is widespread since in these institutions patients with dementia presenting such disorders range between 40 and 50% (Hersch and Falzgraf, 2007). In these contexts, the presence of cognitive, behavioral, and emotional problems is very common, has a direct impact on the well-being of patients and caregivers and, although there has been considerable debate (Angelini et al., 2007), is at the basis of the extensive use of psychotropic drugs. Behavioral and emotional disturbances in people with cognitive impairment are in fact a very important issue in terms of human and financial costs and they have been the focus of several studies (e.g., Doody et al., 2001; Soto et al., 2008).

Special Care Units for Alzheimer’s disease are dedicated to the management of patients with dementia within residential care facilities and, since they are specialized care units, they are ideal contexts for the development and implementation of clinical interventions aimed at improving the management of behavioral problems and enhancing non-pharmacological treatments (Cohen-Mansfield et al., 2010, 2012). The new IPA guidelines (International Psychogeriatric Association, 2012) for the treatment of behavioral and psychological disorders in patients with dementia recommend the use, in combination with drugs, of non-pharmacological interventions that can take into account the person’s history, interests and capabilities. Doll therapy as a non-pharmacological intervention for people in advanced stage of dementia is in line with these guidelines, and its benefits extend to other aspects of the person’s life (behavior, mood, emotion, cognition, affectivity, and sociality) as suggested by several works (e.g., James et al., 2006; Ellingford et al., 2007; Mackenzie et al., 2007). Like any treatment, Doll therapy is not always suitable for everyone. According to the Guidelines for use of dolls and mechanized pets as a therapeutic tool (retrieved March 10, 2014, from www.fightdementia.org.au), dolls are proposed to patients after a careful evaluation considering the personal history, any traumatic events and parenting style. The nursing staff is made aware of the importance of paying attention to the way the person with dementia considers the doll when presented and validates the meaning it has for patient. The nursing staff asks the patients to take care of the doll day by day and this promotes the building of a relationship between patient and doll, which is very useful to prevent disruptive behaviors such as agitation, wandering, anxiety or reduce them in the moment they are emerging. When introducing a doll, it is important to take into account the family’s viewpoint as well as the person with dementia.

Doll therapy as a care tool has been integrated into the context of long-term care institutions, in which the need to find solutions to cognitive, behavioral, and emotional problems showed by people with dementia meets the primary objective of developing good care practices focusing on patients and their needs. One of the first works addressing this issue (Moore, 2001) offers several considerations derived from the observation of behaviors adopted by this kind of patients toward dolls given them by their professional caregivers in an institutionalized context. In particular, this work reported reduction of disturbing behaviors (agitation, aggressiveness, wandering), increased communication between patients and caregivers due to the fact that the doll stimulated conversation on affective topics related to motherhood and caregiving. Furthermore, changes in the caregivers’ attitude toward the dolls (i.e., they treated dolls like real children in the presence of patients) were also reported.

Observational studies in residential contexts confirm these results. For example, Tamura et al. (2001) observed that the signs of emotional discomfort displayed by patients with dementia (i.e., frustration and agitation) decreased in response to the proposal of using a doll. Authors reported that patients seemed more smiling and expressive, they communicated more easily with others and they seemed more cognitively active. Mackenzie et al. (2006) conducted a study with 37 institutionalized patients with dementia who had been observed while interacting with dolls. The observations recorded by the nursing staff during these interactions support the use of a doll as an effective strategy in reducing wandering and oppositional behaviors during service as well as in improving communication between patient and nurse. Similar results were obtained by Ellingford et al. (2007) in a retrospective analysis of the case notes of nursing home residents with dementia 3 months before and after introduction of the Doll therapy. These authors found an increase in doll users’ positive behavior following the introduction of the dolls and a reduction in negative behaviors and aggression.

Doll therapy also offers relevant advantages in terms of costs and benefits: it is an intervention that does not necessarily require the presence of a skilled therapist, as opposed to other non-pharmacological treatments (such as pet psychotherapy, music therapy, art therapy) but can be carried out by different professionals (nurses, educators) once appropriately trained and supervised. Moreover, costs and timing for the implementation and continuation of this kind of treatment are much less demanding than those of other non-pharmacological interventions, psychotropic drugs, or physical restraints.

## THEORETICAL BACKGROUND AND EXPLANATORY HYPOTHESIS OF DOLL THERAPY

The clinical observation of Doll therapy interventions has highlighted how the person with dementia shifts from requesting care and protection for him/herself – through vocalizations, gestures, crying – to reassuring the doll, which is perceived and treated as a real baby. It promotes moments of peacefulness and tranquility, with significant reductions of disruptive behaviors. Patients often display caregiving (rocking, caressing, kissing, squeezing the chest, arranging clothes, combing) and exploratory (manipulating, moving, carefully observing, sniffing) behaviors associated with emotional expressions, such as joy, surprise, tenderness, and concern. In some cases, patients show the desire to feed the doll, change its clothes or to put it to bed, as if they recalled automated responses to the children’s typical needs and were enabled to display them.

Why do people with dementia calm down and reduce attachment requests in response to looking after the doll? Why is Doll therapy effective for some people and not for others, despite similar diagnostic conditions? Today, available knowledge is still limited and there is no unique explanatory model for this intervention; however, some observations begin to emerge and they focus on the meaning patients attribute to the doll and on the interpretation of displayed behaviors as a search for security. In particular, the role that the attachment and caregiving relationship plays in the interplay with the doll is strongly emerging.

The interest in the attachment styles of these patients started to grow and consolidated within the shifting of the theoretical pattern of dementia: from a medical perspective to a bio-psycho-social one. This change not only brought the disease (functional losses) back in the spotlight but also highlighted the skills that the person with dementia maintains, such as the ability to establish relationships with its past and present relevant other. In the recent years, in opposition to the dominant biomedical view, which tends to overlook the existence of the inner world in the person with dementia, contributions emphasizing individual differences, life experiences influencing the reaction to the disease and its course (Stokes, 2000; Ploton, 2010) and the centrality of the individual as embodied subject are growing (Miesen, 1993, 2006; Kitwood, 1997). Bowlby’s (1969, 1988) attachment theory has been developed from a cognitive point of view and defines attachment as an emotional bond with a specific person that is enduring across time and space. Its biological function is self-preservation, which manifests itself in situations of vulnerability (i.e., when one feels scared, sick, tired) through behaviors that are aimed at maintaining proximity to a significant other.

Although Bowlby’s studies on attachment mainly developed from the observation of infant behavior, the author’s statement that attachment representations have an influence “from the cradle to the grave” lays the foundation for the study of these processes along the entire life, old age included (Bowlby, 1979, 1988; Van Assche et al., 2013). In this stage of life, in fact, attachment is of fundamental importance in consideration of the intrinsic aspects of personal vulnerability and the more frequent occurrence, especially in cases of dementia, of experiences of loss and separation (Bradley and Cafferty, 2002; Cosedine and Magai, 2003). The use of attachment theory as the key to the demented patient’s behavior has been particularly developed by Miesen (2010, p. 475), who describes the experience of a person with dementia as “a battle against powerlessness, disruption of daily existence and emotional collapse, similar to the basic reaction of anyone after a trauma of any nature or impact”. With these words, the author explains the consequences arising from deficits in executive and instrumental functioning, which lead to progressive loss of the ability to integrate feelings, thoughts, and emotions in an ongoing view of oneself and the world around him/her. The experience of spatial and temporal disorientation, the misrecognition of familiar faces and places, the progressive inability of verbal expression and comprehension, and many other impairments lead to dependence and isolation. In a state of complete lack of reference points and non-recognition of oneself and of other significant individuals, dominated by emotions of anxiety and fear, seeking proximity and safety is a normal reaction to an abnormal situation (Miesen, 2010). The most frequent expressions such as repetitive questioning, weeping, physical contact requests, following another person in all his movements (shadowing), wandering and complaining, as well as aggressive gestures and mental and physical agitation, may represent forms of attachment requests. When such behaviors occur intensely and frequently they are called “behavioral disorders” (Perren et al., 2007), following the idea previously proposed by Wright et al. (1995) that the dementia-related behavioral problems can be interpreted as attachment behaviors. Therefore, it seems that the challenge for the person with dementia is the continuous search for meaning. Consistent with recent studies (Miesen, 2010; Bisiani and Angus, 2012) we can assume that Bowlby’s theory of attachment represents a possible key to explain the effectiveness of Doll therapy. In a recent study on a patient in a long-term care institution, Bisiani and Angus (2012) claimed that Doll therapy can be used as a therapeutic tool in response to the needs of attachment because it allows patients to experience emotions which have been felt in the field of past significant relationships (with parents), thus bringing the person back to a time in which the request for protection and security was answered. Grounded in several clinical observations, the hypothesis that we here propose is that the emotional experience of the person with dementia during Doll therapy should be framed in a broader view, in which caregiving and exploration systems are active together with the attachment one. Implementing a Doll therapy intervention does not consist in just offering a doll; rather it is a more complex operation in which the nurse primarily creates safety conditions so that the person with dementia can come in contact with the doll. This emotional tuning with the patient within a caring relationship could represent a first response to the expressed need for attachment and thus, by creating a situation of greater safety, it could allow other motivational systems, which are active in patient, to manifest and express in the relationship with the doll. Among these motivational systems (i.e., innate-based systems that represent tendencies to act toward specific goals and to pursue certain forms of interaction between organisms and environments) the exploratory one, which aims to gain knowledge of the environment surrounding the individual, and the caregiving one, which is complementary to that of attachment and is oriented to provide care to another co-specific, behaviorally manifest themselves during Doll therapy. In this sense, Doll therapy really represents a “person-centered approach” as advocated by Kitwood (1997) in his theoretical paradigm, because it can create emotional conditions to meet the human needs of the demented patient.

In light of these considerations, we wanted to experimentally verify whether the behavioral patterns of attachment, caregiving and exploration were actually present in patients treated with Doll therapy, by comparing a sample of patients receiving Doll therapy and a group of control participants. The assumptions that the present work wants to prove are the following:

(1) Patients on Doll therapy accept more frequently the doll compared to other non-anthropomorphic objects;
(2) Patients treated with the Doll therapy display more caregiving behaviors toward the doll compared to controls, since they have developed an attachment relationship with the object;
(3) Patients on Doll therapy display more exploratory behaviors toward other objects compared to controls, because they are less active in searching of reassurance and could therefore focus on exploration.

## MATERIALS AND METHODS

Ten patients (9 women and 1 man; age range = 72–94) who are residents of a Special Care Unit for Alzheimer’s disease in an Italian nursing home were recruited. Participants were diagnosed between 2005 and 2010 and have been residing in the Special Care Unit for an average of 3 years (range 2–4). Five patients had already been treated with Doll therapy for at least 24 months (experimental group), while five patients have never been exposed to Doll therapy (control group). Two patients with Alzheimer’s disease and three patients with vascular dementia were enrolled in each group. Participation in this research was preceded by the presentation of the study to the nursing home’s staff and by a discussion with the patients’ relatives, in order to clarify the nature of this work, which did not involve any intervention on patients, nor environmental changes since the study took place in the context of daily life.

**FIGURE 1:**
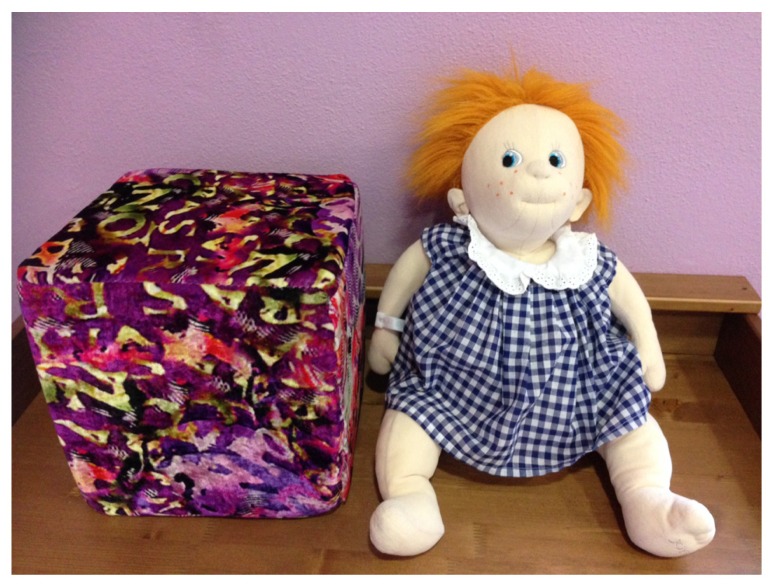
**A doll and the soft foam rubber cube used during the experimental sessions**.

Once obtained the consent, a relative or a support administrator (in case one had been appointed) signed an informed consent form specially arranged and approved by the local ethics committee. Recruited patients were selected in accordance with the opinion of the referring physician and in accordance with the experimental situation. The procedure is not harmful and presents no danger to the physical and psychological health of patients; in the event that a patient would show signs of distress or discomfort (through verbalization or non-verbal cues), the procedure would be immediately suspended. Inclusion criteria were: to be at least 70 years of age; a diagnosis of Alzheimer’s or vascular dementia with a severe cognitive impairment; the presence of behavioral disorders and a minimum ability to understand simple messages and to produce sentences. Exclusion criteria were: a MMSE score above 15; a diagnosis of non-Alzheimer’s or vascular-type dementia, global aphasia, and sensory deficits (vision and hearing). The participants underwent 10 experimental sessions each (two participants performed less experimental sessions for contingent events, i.e., transfer to another nursing home and death); 5 sessions involved the presentation of a doll among those used for Doll therapy and the other 5 sessions involved the presentation of an non anthropomorphic object, i.e., a soft foam rubber cube covered with a colored and velvety textile (see **Figure 1**). The dolls are produced by a Swedish brand and they show features designed to recreate the sensation of touching, looking and holding a child in the arms. The doll and the cube were presented in random order.

Experimental sessions were held inside the residential complex in a room known to patients, with a bed and a chair on which the participant seated; the only people in the room were a nurse and the researcher who handled video recording, who never interacted with the patient and was not visible to him/her since he remained behind a closet.

The experimental protocol consisted of 10 non-consecutive sessions that were conducted over 30 days. The procedure about the interaction between the nurse and the patient and the presentation of the object (the doll or the cube) was the following: when the nurse accompanied the patient in the room and the patient took a seat, the nurse went out and come back with the doll or the cube. The nurse put the doll or the cube in front of the patient and said “Good morning Mr./Mrs.... look.” The nurse gazed at the patient. The tone of voice was quiet. The doll or the cube was showed in the same way: they were hold with both arms in front of the patient and far away from the body. If the patient did not take the doll or the cube at the first attempt, s/he was invited to a second command “Take it” and after waiting a few seconds “Is for you”. If the patient did not take the object after the second request, the nurse did not insist, she went away and said: “I have to go, goodbye Mr./Mrs. ….” If the object was taken, the nurse did not make any comment and did not interact with the patient but she went away from the patient and said “I have to go, goodbye Mr./Mrs. ….” During this procedure the gaze is always upon the patient.

Formally speaking, this procedure involved five standard steps:

(1) A nurse (whom the patient knew) accompanied the patient in the room and invited him/her to sit on the chair.
(2) The nurse presents the object (doll or cube in randomized order) to the patient.
(3) The nurse left the room leaving the patient alone with the object/stimulus.
(4) Interaction with the object: it lasted 3 min starting from the moment when the nurse left the room. This phase was interrupted if patients dropped the object before the time limit.
(5) The nurse returned into the room and took back the object.

The protocol was structured with the goal of recreating a situation of (1) separation from a known figure and (2) interaction with the environment in order to partially recreate the prototypical phases of the “Strange situation” (Ainsworth et al., 1978). The protocol has been simplified and adapted in order to make it administrable to institutionalized patients with dementia and to highlight the interaction behaviors of the elderly with the offered items.

All sessions were videotaped and analyzed through an observational grid specifically developed for this work. The observational grid shows six areas that focus on different kinds of behavioral responses in a dichotomous way (behavior: present/absent) and are detailed in **Table 1**.

**Table 1:** Classification criteria used in the video recording analysis of the protocol.

Area	Classification criteria
Patient’s eye gaze direction at the moment of delivery (on the object/between object and nurse).	Patients keeps her/his eyes fixed on the object; Patient moves her/his eyes from the object to the nurse or vice versa.
Patient’s response at the moment of delivery (s/he accepts/refuses the object).	Acceptance includes: keeping the object in her/his hands for at least 10 s or place it on her/his legs. Refusal includes: not accepting the item, not holding it for at least 10 s, dropping it, returning it to the nurse within 10 s, avoiding visual and tactile contact with the object.
Separation from the nurse (patient accepts it/complains/do not pay attention to the nurse).	Patient accepts: s/he does not recall the nurse’s attention with gestures or vocalizations. Patient protests: s/he cries, calls the nurse with vocalizations or gestures. Patient do not pay attention: s/he shows no changes in eye gaze direction or vocalizations/signs of protest.
Display of exploratory behaviors toward the object (yes/no); if yes, please report their duration in seconds.	Behaviors such as observing the object for at least 5 s, manipulating it, moving it, smelling it, moving it from one hand to the other were classified as exploratory behavior.
Display of caregiving behaviors toward the object (yes/no); if yes, please report their duration in seconds.	Behaviors such as caressing the object, hugging it, rocking it, talking to it, smiling were classified as caregiving behaviors.
Object abandonment (yes/no).	Actions such as dropping the object, placing it on the bed or on the floor, stopping the interaction with it, or manipulating it were classified as object abandonment.

The encoding of video recordings has been carried out separately by two judges: the first one took part in the drafting and implementation of the protocol, while the other was independent and unaware of both the objective of the study and group assignment. To verify whether the assessment was reliable, the level of agreement between the two judges was measured on three dichotomous variables (behaviors of exploration, caregiving, and abandonment). The level of concordance, evaluated with Cohen’s *K*, was found to be as good as indicated by the following values: exploration *K* = 0.522, caregiving *K* = 0.769, abandonment *K* = 0.888 (*p* < 0.001 in every case). For our analysis, we only used data provided by the blind referee as he was considered uninfluenced by potential expectations about the study.

At the baseline assessment, some neurological and cognitive indicators were measured: Tinetti scale for balance assessment (Tinetti et al., 1986), Barthel scale for the level of autonomy (Mahoney and Barthel, 1965), and NPI scale for behavioral symptoms (Cummings et al., 1994) were administered in addition to MMSE (Folstein et al., 1975).

Statistical analyses were performed using “chi square” analysis and “*t*-test” for independent samples, as appropriate; all analyses were performed with SPSS software (SPSS version 20.0, IBM, Chicago, IL, USA).

## RESULTS

We found no significant differences between the two groups in age, education, time of institutionalization, number of family visits or any other baseline variable (see **Table 2**); both experimental sample and control group showed a high risk of falls, a severe lack of autonomy in the activities of daily living and a high level of behavioral problems.

**Table 2:** Demographic and clinical characteristics.

	Experimental group Mean ± SD	Control group Mean ± SD	*t*-Student	*p* Value
Age	85.8 ± 7.3	83.6 ± 7.4	0.473	0.649
Years of education	8.8 ± 2.9	6.2 ± 1.6	1.755	0.117
Months in institution	38.8 ± 13.7	32.6 ± 13.9	0.710	0.498
Family visits per week	4.2 ± 1.8	5.4 ± 1.5	1.145	0.285
MMSE	5.2 ± 4.7	4.6 ± 5.7	0.182	0.860
NPI	21.8 ± 13.3	21.2 ± 7.4	0.088	0.932
Barthel	19 ± 11.9	23.4 ± 11.1	–0.604	0.563
Tinetti	13.2 ± 8.5	12.8 ± 5.7	0.088	0.933

*Barthel, Barthel scale for the level of autonomy; MMSE, Mini Mental State Examination; NPI, Neuropsychiatric Inventory; Tinetti, Tinetti scale for balance assessment.*

### STEP 1: PRESENTATION OF THE OBJECT

At the presentation of the object, eye gaze of participants has been observed to verify if the two objects (doll or cube) acquired a different communicative and relational value; it was then observed whether the participants maintained their gaze on the presented object or if they shifted it between object and nurse. We considered this last behavior as an indicator of a relational dimension.

Globally considering the presentation of the two objects, results showed a significant difference between experimental and control participants, with respect to gaze direction; more specifically, patients treated with Doll therapy were more likely to shift their gaze between object and nurse compared to control patients (χ^2^ = 5.959, *p* = 0.015). Considering the presentation of the two objects separately, there were no significant differences: experimental participants tended to shift gaze between object and nurse with equal frequency for both doll and cube (χ^2^ = 0.811, *p* = 0.368), while the control group kept the gaze fixed on both objects with the same frequency (χ^2^ = 0.201, *p* = 0.654).

In this step, all patients equally accepted both objects (doll and cube) with no significant differences between experimental (χ^2^ = 0.296, *p* = 0.587) and control group (χ^2^ = 0.170, *p* = 0.680).

### STEP 2: SEPARATION FROM THE NURSE

In this phase, results showed a significant difference in the behaviors of the participants assigned to the two groups; in particular, patients from the experimental group accepted separation from the nurse with greater frequency, while patients of the control group pay little attention to the moment of separation from the nurse and show less changes in eye gaze direction or vocalizations/signs of protest (χ^2^ = 13.740, *p* = 0.001). Moreover, there were no differences in patients’ response to separation on the basis of the delivered object for both experimental (χ^2^ = 1.213, *p* = 0.545) and control group (χ^2^ = 2.131, *p* = 0.345).

### STEP 3: INTERACTION WITH THE OBJECT

With regard to the interaction behaviors with the object, it has been observed that the participants of the experimental group tended to display both behaviors of exploration (χ^2^ = 3.960, *p* = 0.047) and caregiving (χ^2^ = 12.072, *p* = 0.001) more frequently compared to the control group.

By separately assessing behaviors shown toward the two objects by each group, results pointed out a significant difference: experimental patients showed greater frequency of cube exploration behaviors compared to those directed at the doll (χ^2^ = 5.137, *p* = 0.023); moreover, the same patients tended to display caregiving behaviors more frequently toward the doll (χ^2^ = 35.368, *p* < 0.001).

In contrast, control patients explored the two objects with the same frequency (χ^2^ = 0.271, *p* = 0.603) and there were no significant differences in caregiving behaviors, which were quite infrequent for both the cube and the doll (χ^2^ = 1.365, *p* = 0.243).

**FIGURE 2:**
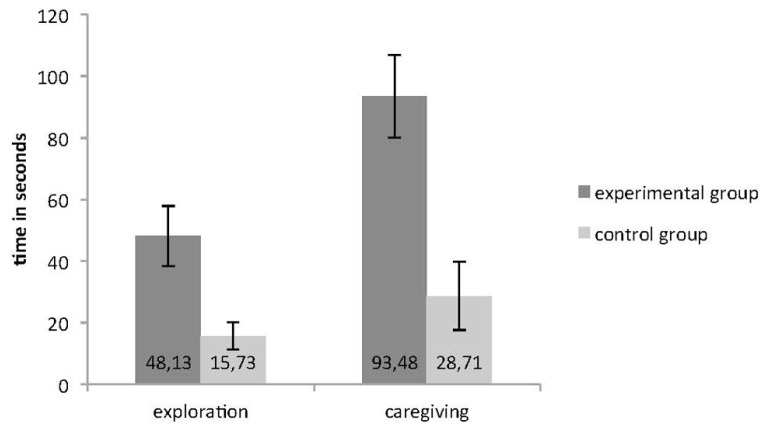
**Time in seconds spent by the participants of the two groups in object exploration and caregiving with error bars representing standard errors**.

The time in seconds that patients spent in exploration and caregiving behaviors was measured and results showed that experimental patients explored and looked after the objects not only with greater frequency, but also for a significantly greater amount of time (exploration: *t* = 3.033, *p* = 0.005; caregiving: *t* = 2.655, *p* = 0.013; see **Figure 2**).

### STEP 4: SEPARATION FROM THE OBJECT

Regarding the separation from the object (doll or cube), we assessed whether participants interrupted the contact with the object or kept it: no significant difference was found in the frequency of abandonment behaviors (χ^2^ = 0.688, *p* = 0.407). However, when considering the two groups separately, our data demonstrated that experimental patients abandoned the cube with a significantly higher frequency than the doll (χ^2^ = 17.094, *p* < 0.001); on the contrary, control patients left the two objects with the same frequency (χ^2^ = 0.016, *p* = 0.900).

## DISCUSSION

To the best of our knowledge, the present one is the first study aimed to assess the effects of Doll therapy in which a group of treated patients with dementia has been compared with a control group. The results provide some important data to support the effectiveness of Doll therapy in promoting and maintaining the affective-relational dimension of attachment-caregiving and the attentive dimension of exploration in patients with advanced stage of dementia.

Participants of the experimental group were found more interested in the relational value of the doll as it can be derived from the eye gaze direction, which alternated between the object and the nurse, in contrast to control participants who focused their attention mainly on the object. Furthermore, participants treated with Doll therapy for at least 2 years showed a preference for the doll and implemented exploration behaviors to a greater extent toward the cube as a mainly instrumental object that did not have any relational value. This aspect also emerged clearly from the video recordings in which the uncertainty expressed by patients presented with the cube and their search for meaning through questions addressed to the nurse were observed. We interpreted these data considering that the doll represented a known situation for these patients and that the cube activated their curiosity and thus the need/desire for exploration. Interestingly, exploration was more frequent and had longer duration; therefore we hypothesize a possible enhancement of the participants’ attentive ability when they were in a relational context perceived as safe, such as the one of Doll therapy. On the contrary, participants of the control group showed a significant indifference to both stimuli toward which they behaved similarly, without showing any clear preference and in general displaying few exploration or caregiving behaviors toward them. This behavior has been interpreted as a non-allocation of relational significance to the doll that lead to the non-activation of the caregiving system, which requires the recognition on the doll, conceptualized as an anthropomorphic stimulus, as a relational subject (Feneey and Collins, 2001; Mikulincer et al., 2001).

Concerning the low frequency of exploration behaviors displayed toward the two stimuli by control participants, it is interesting to note that, according to the attachment theory, the ability to explore arises as a consequence of the perception of a safety state, which control participants did not seem to have (e.g., Ardito et al., 2004; Steele et al., 2004; Cookman, 2005; Adenzato et al., 2006; Cicerale et al., 2013). In particular, according to the attachment theory humans are motivated to maintain a dynamic balance between familiarity-preserving, stress-reducing behaviors (attachment to protective individuals and to familiar home sites, retreat from the strange and novel) and antithetical exploratory and information-seeking behaviors (Bretherton, 1992). Recently, Tops et al. (2013, 2014) have proposed a neuroendocrinological update of the attachment theory according to which in forming close and supportive relationships people shift from novelty seeking to preference of social familiarity through a process mediated mainly by the oxytocin, a neuropeptide which may be involved in the overlapping mechanisms of stable attachment formation and stress coping. In line with this proposal, we speculate that during a Doll therapy the progressive exposure, interaction, and familiarity with the doll contributes to the formation of the attachment relationship and, consequently, to the reduction of the behavioral and emotional disturbances often observed as a consequence of this intervention in people in advanced stage of dementia.

The fact that behaviors of exploration and caregiving displayed by experimental participants persist even after at least 2 years from the beginning of Doll therapy suggests that it represents an intervention that allows to build and keep a significant relational situation with the doll over time, thus highlighting relational skills that are generally compromised in these patients. In addition, we used the same doll for all treated patients, which is unusual because each patient usually receives a doll with different color of hair, eyes and clothing. This again suggests that patients perceive and recognize the anthropomorphic and relational aspects of it instead of the mere physical characteristics of the object (e.g., hair color).

## LIMITATIONS AND CONCLUDING REMARKS

This paper presents some methodological limitations that should be underlined. Certainly, the sample size of the study is extremely limited, even considered the difficulty of collecting a wider number of patients in this type of intervention. Due to the exploratory nature of the study, we recruited only few patients in order to set up an experimental setting that was as much as possible under our control, but obviously future studies should aim at recruiting a larger number of patients in order of seeing whether our preliminary results can be confirmed and hopefully corroborated on a large scale. Second, only observational indices were used because of a lack of standardized tools for assessing affective-relational dynamics of attachment-caregiving in patients with dementia; however, the use of a categorical scale allowed us to obtain a reliable assessment. Third, we recognize that Doll therapy studies have been considered ethically controversial by some authors. According to them, the use of dolls is infantilizing for patients who end up being treated like children (Hughes et al., 2006). Others argue that the use of dolls would be ethically questionable because caregivers use an illusion to lead patients to believe that they are relating with real children (Cayton, 2001), and indeed delusional misidentification due to neurodegenerative processes in brain areas associated with mental states attribution (Bara et al., 2011; Cavallo et al., 2011; Adenzato et al., 2012; Poletti et al., 2012; Adenzato and Poletti, 2013) has been reported in people with dementia (e.g., Molchan et al., 1990; Shanks and Venneri, 2002; Van den Stock et al., 2013). These considerations directed our reflection on the ethical implications of Doll therapy, since we believe that the dignity and self-determination of people with dementia should be protected. However, we agree with the majority of authors who believe that presenting a doll to people with dementia does not mean lying to them or deceiving them. Instead, we consider the broader meaning of this gesture and the fact that the individual him/herself will decide whether it is a doll or a real baby; caregivers will simply confirm this perception (Andrew, 2006).

Despite the limitations of this study, our findings show new opportunities to enable good practice in relation to people with dementia. Our specific interest is aimed at individuals who are in a stage of disease in which relationships become difficult and painful due to the difficulty the nurses and patients themselves encounter in reciprocal interpersonal and emotional tuning. This phenomenon may cause an increase in behavioral and psychological disorders. Our results seem to show that in these patients, together with the activation of the attachment system, when in a situation of reassurance offered by Doll therapy, some of them may experience a re-activation of the system of caregiving and of exploration.

Summing up, it seems that the emotional experience of Doll therapy promotes improvements in the ability to relate with the surrounding world that persists over time and is clinically significant. This may be important for managing and caring for patients with dementia in institutionalized context.

## Conflict of Interest Statement

The authors declare that the research was conducted in the absence of any commercial or financial relationships that could be construed as a potential conflict of interest.
